# Age‐related differences in MRI signal intensity of the quadriceps and patellar tendons: Implications for ACL graft selection

**DOI:** 10.1002/jeo2.70617

**Published:** 2026-01-08

**Authors:** Takuya Kinoshita, Yusuke Hashimoto, Masatoshi Hoshino, Kentaro Inui, Takeshi Sugimoto, Shinji Takahashi, Hidetomi Terai

**Affiliations:** ^1^ Department of Orthopaedic Surgery Osaka Saiseikai Nakatsu Hospital Osaka Osaka Prefecture Japan; ^2^ Department of Orthopaedic Surgery Osaka Metropolitan University Graduate School of Medicine Osaka Japan; ^3^ Department of Health and Sport Management Osaka University of Health and Sports Science, Graduate School of Sport and Exercise Science Osaka Japan; ^4^ Department of Orthopaedic Surgery Osaka Global Orthopedic Hospital Osaka Japan

**Keywords:** age‐related changes, anterior cruciate ligament reconstruction, graft choice, MRI signal intensity, quadriceps tendon, tendon thickness

## Abstract

**Purpose:**

To compare the thickness and magnetic resonance imaging (MRI) signal intensity at the patellar attachment of the quadriceps tendon (QT) and patellar tendon (PT) across age groups in order to evaluate age‐related degenerative changes relevant to graft selection for anterior cruciate ligament (ACL) reconstruction. It was hypothesised that older groups would show increased signal intensity.

**Methods:**

Patients aged 14–65 years who underwent knee MRI were retrospectively reviewed. Participants were categorised into the senior (50–65 years), middle (30–49 years), young (20–29 years) and teen (14–19 years) groups. After propensity score matching for sex, height and weight, 64 participants were included in each age group. T2* sagittal MR images of the ACL graft harvest area were used to measure the tendon thickness and signal intensity.

**Results:**

QT was significantly thicker and had lower signal intensity than PT across all age groups. Thickness did not significantly differ between QT and PT across age groups. QT signal intensity was significantly higher in seniors than in young adults, whereas PT signal intensity was significantly higher in both middle‐aged and senior groups than in the young group.

**Conclusions:**

The QT was thicker than the PT and exhibited a lower signal intensity across all age groups. Compared to the 20–29‐year age group, signal intensity was higher in the 50–65‐year age group for the QT and in the 30–65‐year age group for the PT. These MRI‐based findings suggest that the QT may offer advantages as a graft source, particularly in patients aged >30 years.

**Level of Evidence:**

Level III, cross‐sectional study.

**Abbreviations**
ACL: anterior cruciate ligament
ACLR: anterior cruciate ligament reconstruction
ICC: interclass correlation coefficient
MRI: magnetic resonance imaging
PCL: posterior cruciate ligament
PT: patellar tendon
QT: quadriceps tendon

## INTRODUCTION

Although the optimal choice of graft for anterior cruciate ligament (ACL) reconstruction (ACLR) remains controversial, the quadriceps tendon (QT) has attracted increasing attention as a promising alternative in recent years [12, 16]. Notably, QT grafts show no difference in clinical outcomes and similar or superior graft survival rates as compared to patellar tendon (PT) and hamstring grafts; however, QT grafts are associated with reduced donor site morbidity compared to PT grafts [5, 8, 10, 13, 17, 19, 20, 27, 29, 30]. With the growing population of highly active middle‐aged and older individuals, the incidence of ACLR in these age groups has been increasing [25]. With aging, tendons become more susceptible to degeneration, increasing the risk of tendinopathies and injuries [18, 21]. Particularly, the PT exhibits reduced tensile strength and increased laxity with age [7, 31, 37], necessitating the consideration of age for graft choice. Previous reports have indicated higher failure rates in ACLR using PT grafts and allografts in the presence of tendinopathy [3, 23] and in older patients, respectively [33]. These findings highlight that degenerative change of the graft is a crucial factor. A previous study demonstrated that magnetic resonance imaging (MRI) T2* signal intensity is associated with the maximum failure load and stiffness of the graft tendon [6]. MRI signal intensity is used to evaluate ACL graft maturation, with higher signal intensity suggesting tendon degeneration [2, 31, 35]. Moreover, a significant correlation is observed between MRI and intraoperative measurements of both QT and PT thickness [1, 34], and MRI signal changes in jumpers' knees are reportedly correlated with degenerative findings in pathological tissue samples [36].

Graft thickness is another important factor influencing graft survival. In hamstring tendon grafts, smaller diameters have been associated with higher failure rates [9]. Previous studies have reported that the QT is thicker than the PT; [26] however, to the best of current knowledge, no study has investigated graft thickness stratified by age.

This study aimed to compare the thickness and MRI signal intensity of QT and PT at their patellar attachment sites across different age groups to elucidate the characteristics of each age group. It was hypothesised that older age groups would show increased signal intensity on MRI.

## MATERIALS AND METHODS

### Ethics approval

This retrospective cross‐sectional study was approved by the Ethics Committee and Internal Review Board of Osaka Saiseikai Nakatsu Hospital (approval no.: 2024‐19).

### Study population

**Figure 1:**
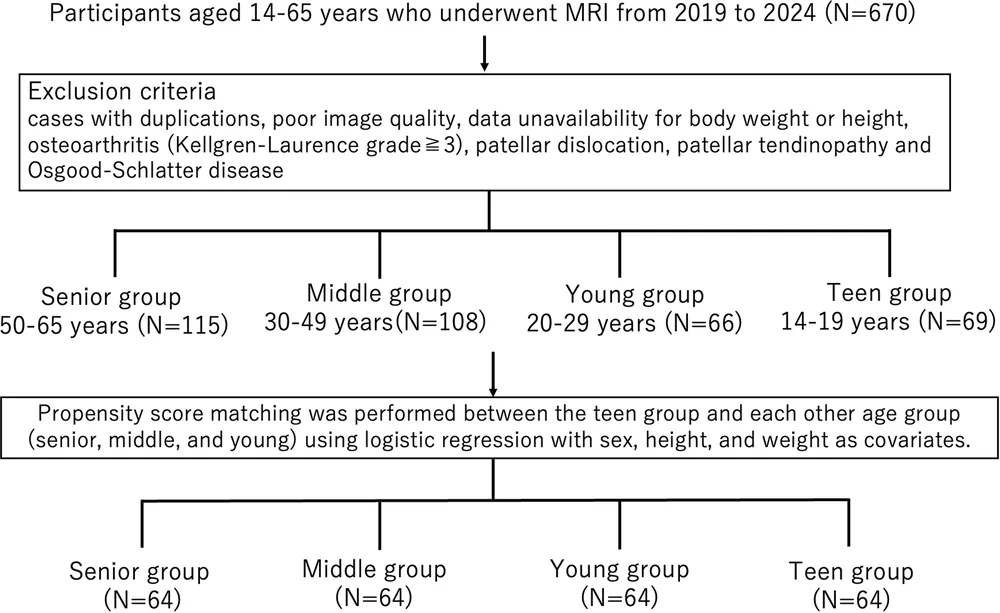
Flowchart of participant enrolment. A total of 670 patients aged 14–65 years who underwent magnetic resonance imaging were included in the study. After applying the exclusion criteria, the patients were categorised into four age groups. Then, propensity score matching was performed, resulting in 64 patients per group for the final analysis.

The study included 670 knees of participants aged 14–65 years who underwent knee MRI at the hospital between 2019 and 2024. The flowchart of the study is shown in Figure 1. The exclusion criteria were duplicate entries, poor image quality, unavailability of data on body weight or height, osteoarthritis (Kellgren–Lawrence grade ≥3), patellar dislocation, patellar tendinopathy, and Osgood–Schlatter disease. The participants were categorised into four groups: senior (50–65 years), middle (30–49 years), young (20–29 years) and teen (14–19 years) groups. To adjust for potential confounding factors, we performed separate 1:1 propensity score matching between the teen group and each of the other three groups (senior, middle and young groups) using nearest‐neighbour matching. Propensity scores were estimated using logistic regression models, including sex, height and weight as covariates.

### Radiography analyses

**Figure 2:**
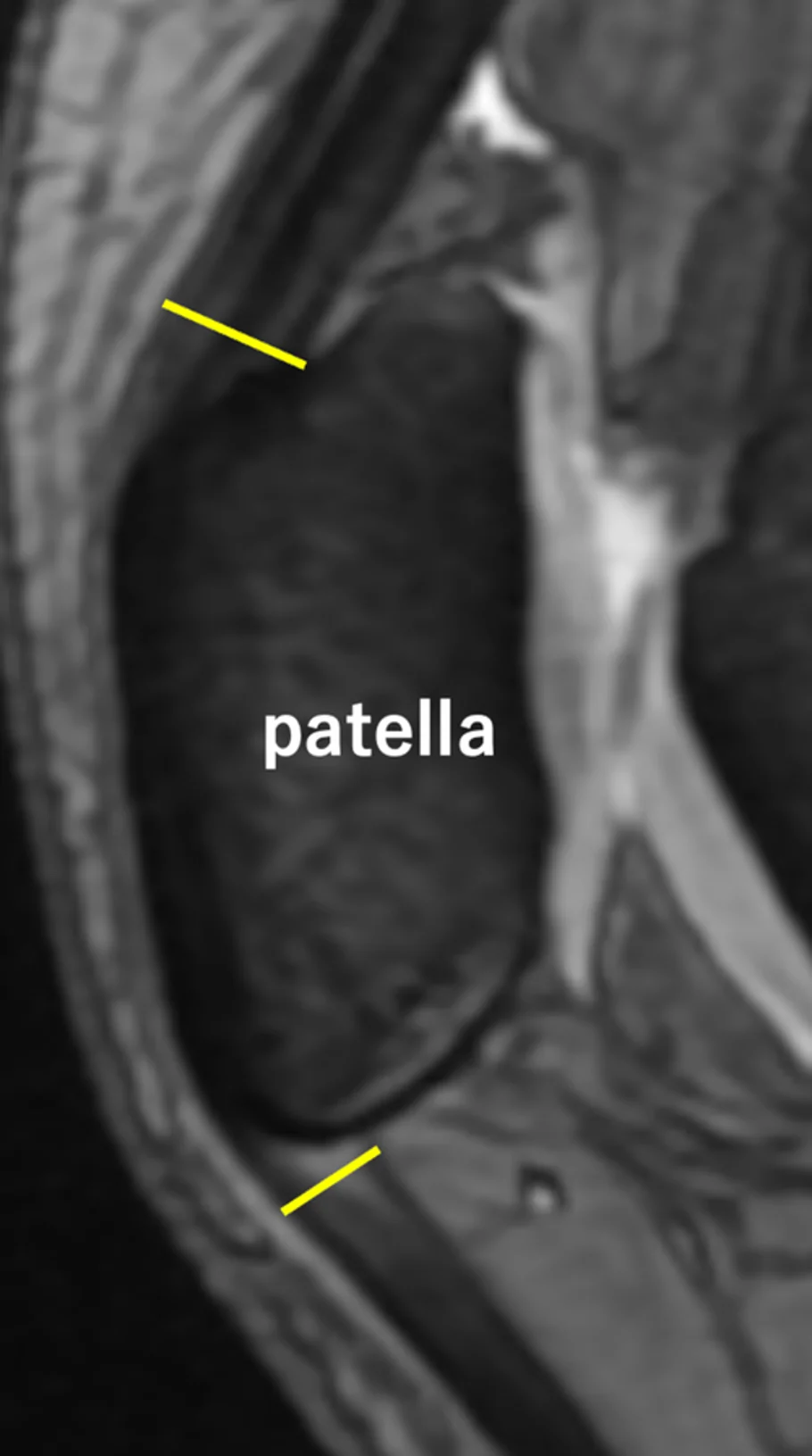
Measurement of tendon thickness. Sagittal magnetic resonance imaging showing the measurement of quadriceps tendon and patellar tendon thicknesses at potential anterior cruciate ligament graft harvest sites. The yellow lines indicate the thickness measurement locations of the quadriceps tendon (upper line) and patellar tendon (lower line). These were measured perpendicular to the tendon at their patellar attachments.

**Figure 3:**
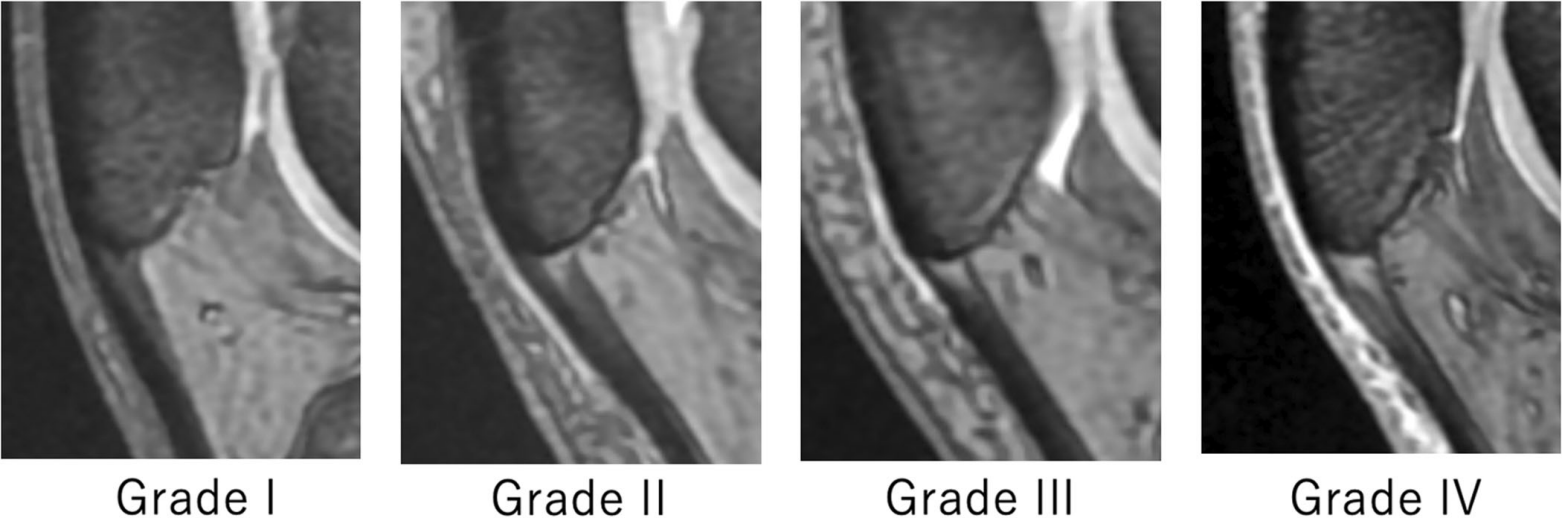
Grading system for tendon MRI signal intensity. Representative sagittal MR images of the quadriceps and patellar tendons at their potential ACL graft harvest sites demonstrate the four‐grade classification system for the signal intensity, based on previous studies. Grade Ⅰ (homogeneous low signal intensity), Grade Ⅱ (high signal intensity regions comprising <50% of the area), Grade Ⅲ (high signal intensity regions comprising ≥50% of the area), and Grade Ⅳ (diffuse high signal intensity). ACL, anterior cruciate ligament; MRI, magnetic resonance imaging.

MRI was performed using a 3.0‐Tesla MR imager (Achieva 3 T X series; Philips Healthcare, Best, Netherlands) with a 16‐cm field of view, number of excitation of 2, 1.50‐mm slice thickness, and 1.65‐mm slice spacing. All MR images were acquired using a standardised sagittal imaging protocol. The thickness and signal intensity of QT and PT were measured at the deepest layer in their patellar attachment sites, corresponding to the typical graft harvest sites for ACLR, using T2*‐weighted sagittal MR images (Figure 2) [11, 24]. Signal intensity was categorised into four grades according to previous reports. The posterior cruciate ligament (PCL) was used as an internal reference, with signal intensity equivalent to that of the PCL defined as normal (Grade I). Signal intensity was graded as follows: Grade II, high signal intensity involving <50% of the tendon area; Grade III, high signal intensity involving ≥50% of the tendon area; and Grade IV, diffuse high signal intensity throughout the tendon (Figure 3) [14, 35].

### Statistical analysis

A one‐way analysis of variance was used to compare the thickness among the four groups. Student's t‐test was used to compare the thickness and signal intensity between QT and PT. The Mann–Whitney *U* test was used to compare the signal intensity between the QT and PT. Because signal intensity grade is an ordinal variable, intergroup differences were analysed using the Kruskal–Wallis test, followed by the Steel–Dwass test for multiple pairwise comparisons. Statistical significance was set at *p* < 0.05. Because this retrospective study included all eligible patients who met the inclusion and exclusion criteria, the sample size was not determined by an a priori power analysis. Post hoc power analysis was not performed because statistically significant differences had already been confirmed for the main outcomes. Interclass correlation coefficients (ICCs) were assessed by two fellowship‐trained orthopaedic surgeons, and ICCs were assessed twice by one orthopaedic surgeon, with a 3‐month interval between assessments. The level of agreement was interpreted as follows: ICC values > 0.80 were considered to indicate excellent reproducibility; values between 0.61 and 0.80, good; values between 0.41 and 0.60, moderate; and values ≤ 0.40, poor. Easy R (EZR software, version 1.61) was used throughout the study [15].

## RESULTS

**Figure 4:**
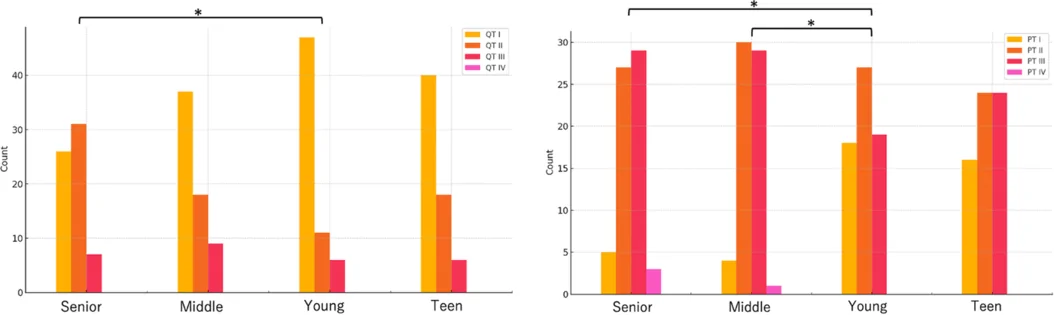
Distribution of MRI signal intensity grades of the quadriceps tendon (QT) (left) and patellar tendon (PT) (right) across the four age groups: Senior, Middle, Young and Teen. Asterisks (*) indicate statistically significant differences among age groups (*p < 0.05). MRI, magnetic resonance imaging.

**Figure 5:**
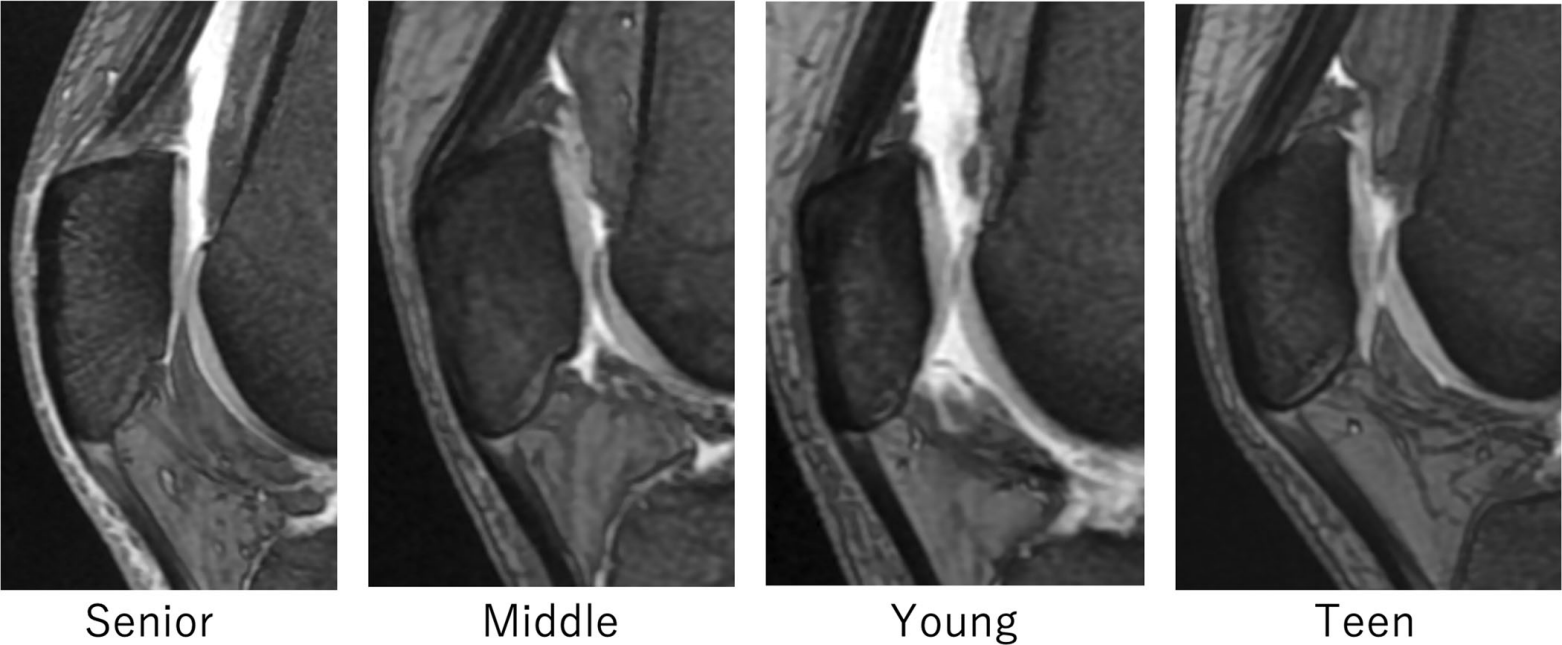
Representative cases of sagittal MR images at potential ACL graft harvest sites. Compared to the young group, QT signal intensity was higher in the senior group, whereas PT signal intensity was higher in both the senior and middle groups. In representative cases, the signal intensity grades were as follows: the senior group (QT: Grade III, PT: Grade III), middle group (QT: Grade I, PT: Grade III), young group (QT: Grade I, PT: grade I), and teen group (QT: Grade I, PT: Grade II). ACL, anterior cruciate ligament; MRI, magnetic resonance imaging; PT, patellar tendon; QT, quadriceps tendon.

Each of the four groups comprised 64 knees (32 male and 32 female knees) (Figure 1). The mean heights were 165.5 ± 8.5 cm, 165.3 ± 9.0 cm, 165.6 ± 9.8 cm and 164.8 ± 7.8 cm in the teen, young, middle and senior groups, respectively. The mean weights were 60.1 ± 11.1 kg, 60.2 ± 11.2 kg, 60.9 ± 14.5 kg and 60.8 ± 12.1 kg, respectively. There were no significant differences in demographic data across the groups (Table 1). QT was significantly thicker and exhibited lower‐grade signal intensity than PT across all groups (Table 2). Although no significant differences in thickness were observed among the age groups for either QT or PT, significant differences were found in signal intensity (Table 2). The distribution of signal intensity grades for the QT and PT across the four age groups is summarised below. For the QT, the numbers of participants with Grades I, II and III were as follows: 40, 18 and 6 in the teen group; 47, 11 and 6 in the young group; 37, 18 and 9 in the middle group; and 26, 31 and 7 in the senior group. For PT, the numbers of participants with Grades I, II, III and IV were: 16, 24, 24 and 0 in the teen group; 18, 27, 19 and 0 in the young group; 4, 30, 29 and 1 in the middle group; and 5, 27, 29 and 3 in the senior group. For the QT, the senior group exhibited significantly higher grade signal intensity than the young group. For the PT, both the middle and senior groups showed significantly higher signal intensity grades than the young group (Figure 4). Representative MR images of each group are presented in Figure 5.

**Table 1:** Demographic data.

	Senior	Middle	Young	Teen	*p* value
Height, cm	164.8 ± 7.82	165.6 ± 9.78	165.3 ± 9.00	165.5 ± 8.50	0.962
Weight, kg	60.8 ± 12.1	60.9 ± 14.5	60.2 ± 11.2	60.1 ± 11.1	0.979

*Note*: Data are presented as mean ± standard deviation.

**Table 2:** Comparison of thickness and signal intensity between QT and PT, and among age groups.

	Senior	Middle	Young	Teen	*p* value
QT thickness, mm	7.47 ± 0.942	7.22 ± 1.01	7.59 ± 0.814	7.54 ± 1.05	0.141
PT thickness, mm	4.78 ± 0.809	4.90 ± 0.908	4.73 ± 0.851	4.77 ± 0.821	0.703
P	<0.001	<0.001	<0.001	<0.001	
QT signal intensity	2 (1–3)	1 (1–3)	1 (1–3)	1 (1–3)	0.009
PT signal intensity	2.5 (1–4)	2 (1–4)	2 (1–3)	2 (1–3)	0.002
*p*‐value	<0.001	<0.001	<0.001	<0.001	

*Note*: Data are presented as mean ± standard deviation for thickness and median (range) for signal intensity grade.

Abbreviations: PT, patellar tendon; QT, quadriceps tendon.

The inter‐ and intra‐observer reliabilities for thickness measurements were 0.901 and 0.915 (95% confidence interval [CI], 0.862–0.929 and 0.881–0.939), respectively, while the corresponding reliabilities for signal intensity measurements were 0.733 and 0.829 (95% CI, 0.641–0.804 and 0.765–0.876), respectively. These results indicated excellent or good reproducibility.

## DISCUSSION

The important findings of the present study are twofold. Across all age groups, the QT was thicker and exhibited lower‐grade signal intensity than the PT. Additionally, compared with the young and teen groups, the QT signal intensity was higher in the senior group, whereas the PT signal intensity was significantly higher in the senior and middle groups. The hypothesis that older age groups would exhibit increased MRI signal intensity was supported by the results, as both the QT and PT demonstrated significantly higher signal intensity in older age groups. Previous studies have reported that patients with a high MRI signal intensity in the PT are associated with higher failure rates in ACLR using PT grafts [3, 23]. MRI signal changes in the jumper's knee are reportedly correlated with degenerative findings in pathological tissue samples [36]. However, as the present study excluded cases of patients with patellar tendinitis, it remains unclear whether the signal intensity changes observed truly reflect tendon degeneration.

In the present study, the QT showed a significantly higher signal intensity in the senior group than in the young and teen groups, while no significant difference was observed between the middle and young or teen groups. Contrastingly, the PT exhibited a significantly higher signal intensity in both the senior and middle groups than in the young and teen groups. These findings suggest that the QT may be a more advantageous graft choice than the PT for ACLR in patients in their 30 s and 40 s. Previous studies on PT allografts have demonstrated that the bone‐tendon junction weakens with age [32], resulting in higher failure rates in individuals aged > 50 years [33]. Consequently, it is recommended to use allografts from donors aged < 40 years [22]. Additionally, PT has been reported to exhibit decreased modulus with advancing age [8]. Contrastingly, a study on ACLR in highly active individuals who were aged > 50 years reported no re‐ruptures and no significant differences in clinical outcomes between QT and hamstring grafts [25].

Previous studies have reported that the QT is thicker than the PT [26], which was observed in the present study. While there have been no prior reports on age‐related thickness differences, the present study found no significant differences in thickness across age groups for either the QT or PT. Although a larger graft diameter is not always preferable due to the risk of impingement, reports on hamstring grafts have indicated that a smaller graft diameter is associated with a higher failure rate [4, 28].

MRI evaluation revealed that the QT maintains greater thickness and lower signal intensity than the PT across all age groups, with relatively preserved quality in middle‐aged individuals. These findings suggest that the QT may serve as a reliable graft option in routine clinical practice, particularly for middle‐aged patients undergoing ACLR. However, as this conclusion is derived solely from imaging assessments, it should be interpreted with caution. Further prospective studies correlating MRI characteristics with intraoperative graft quality and postoperative clinical outcomes are required to determine whether these imaging findings translate into superior clinical performance.

This study has some limitations. First, there was a potential selection bias arising from the inclusion of only patients who underwent MRI. Second, the middle and senior groups may include a higher proportion of individuals who are not actively engaged in sports, limiting the generalisability of the findings to patients with typical ACL injuries. Third, thresholds of clinical relevance, such as the minimal clinically important difference have not been established for MRI signal intensity or tendon morphology. Therefore, the present findings should be interpreted as imaging‐based observations that may provide indirect implications for graft selection, rather than as direct indicators of clinical improvement. Fourth, the present study did not assess the relationship between MRI findings and intraoperative graft quality or patient‐reported outcomes. Finally, tendon signal intensity may have temporarily increased following high‐intensity exercise or knee injury. Because exercise intensity, timing, overall activity level and subclinical factors, such as micro‐degeneration or recent mechanical overload, were not assessed, their potential influence on signal intensity remains unclear.

## CONCLUSION

The QT was thicker than the PT and exhibited a lower signal intensity across all age groups. Compared to the 20–29‐year age group, signal intensity was higher in the 50–65‐year age group for the QT and in the 30–65‐year age group for the PT. These MRI‐based findings suggest that the QT may offer advantages as a graft source, particularly in patients aged > 30 years.

## AUTHOR CONTRIBUTIONS

Takuya Kinoshita conceived and designed the study. Takeshi Sugimoto contributed to data acquisition. Kentaro Inui and Yusuke Hashimoto performed the data interpretation. Masatoshi Hoshino and Hidetomi Terai provided critical revisions and supervised the project. Shinji Takahashi contributed to Statistical analysis and validation. All authors contributed to drafting the manuscript, approved the final version, and agree to be accountable for all aspects of the work.

## CONFLICT OF INTEREST STATEMENT

The authors declare no conflicts of interest.

## ETHICS STATEMENT

This study was approved by the Ethics Committee and Internal Review Board of Osaka Saiseikai Nakatsu Hospital (approval no.: 2024‐19). Informed consent was obtained through an opt‐out method in accordance with the protocol approved by the Ethics Committee. Study information was made publicly available, and patients had the opportunity to refuse participation.

## Data Availability

The data that support the findings of this study are available from the corresponding author upon reasonable request.

## References

[1] Agarwal S , de Sa D , Peterson DC , Parmar D , Simunovic N , Ogilvie R , et al. Can preoperative magnetic resonance imaging predict intraoperative autograft size for anterior cruciate ligament reconstruction? a systematic review. J Knee Surg. 2019;32:649–658. 10.1055/s-0038-1666830 29980152

[2] Aitchison AH , Alcoloumbre D , Mintz DN , Hidalgo Perea S , Nguyen JT , Cordasco FA , et al. MRI signal intensity of quadriceps tendon autograft and hamstring tendon autograft 1 year after anterior cruciate ligament reconstruction in adolescent athletes. Am J Sports Med. 2021;49:3502–3507. 10.1177/03635465211040472 34612708

[3] Alentorn‐Geli E , Gotecha D , Steinbacher G , Álvarez‐Díaz P , Barastegui D , Seijas R , et al. The presence of patellar tendinopathy in the bone‐patellar tendon‐bone autograft may increase the risk of anterior cruciate ligament graft failure. Knee Surg Sports Traumatol Arthrosc. 2019;27:766–772. 10.1007/s00167-018-5066-4 30141146

[4] Alkhalaf FNA , Hanna S , Alkhaldi MSH , Alenezi F , Khaja A . Autograft diameter in ACL reconstruction: size does matter. SICOT‐J. 2021;7:16. 10.1051/sicotj/2021018 PMC7984146 33749586

[5] Barié A , Sprinckstub T , Huber J , Jaber A . Quadriceps tendon vs. patellar tendon autograft for ACL reconstruction using a hardware‐free press‐fit fixation technique: comparable stability, function and return‐to‐sport level but less donor site morbidity in athletes after 10 years. Arch Orthop Trauma Surg. 2020;140:1465–1474. 10.1007/s00402-020-03508-1 PMC7505888 32504178

[6] Biercevicz AM , Miranda DL , Machan JT , Murray MM , Fleming BC . In situ, noninvasive, T2*‐weighted MRI‐derived parameters predict ex vivo structural properties of an anterior cruciate ligament reconstruction or bioenhanced primary repair in a porcine model. Am J Sports Med. 2013;41:560–566. 10.1177/0363546512472978 PMC3593999 23348076

[7] Blevins FT , Hecker AT , Bigler GT , Boland AL , Hayes WC . The effects of donor age and strain rate on the biomechanical properties of bone‐patellar tendon‐bone allografts. Am J Sports Med. 1994;22:328–333. 10.1177/036354659402200306 8037272

[8] Calvert ND , Ebert JR , Radic R . Kneeling tolerance when using quadriceps tendon autograft for anterior cruciate ligament reconstruction is superior to hamstring tendon autograft. Knee Surg Sports Traumatol Arthrosc. 2025;33:2390–2396. 10.1002/ksa.12583 39810716

[9] Conte EJ , Hyatt AE , Gatt CJ , Dhawan A . Hamstring autograft size can be predicted and is a potential risk factor for anterior cruciate ligament reconstruction failure. Arthroscopy. 2014;30:882–890. 10.1016/j.arthro.2014.03.028 24951356

[10] Dai W , Leng X , Wang J , Cheng J , Hu X , Ao Y . Quadriceps tendon autograft versus bone‐patellar tendon‐bone and hamstring tendon autografts for anterior cruciate ligament reconstruction: a systematic review and meta‐analysis. Am J Sports Med. 2022;50:3425–3439. 10.1177/03635465211030259 34494906

[11] Hashimoto Y , Yamasaki S , Iida K , Kinoshita T , Nishino K , Takigami J , et al. Preoperative planning and harvesting technique of quadriceps tendon with bone block for anterior cruciate ligament reconstruction: an evidence‐based, cosmetic, and precise harvest method. Arthrosc Tech. 2025;14:103258. 10.1016/j.eats.2024.103258 PMC11977154 40207332

[12] Heffron WM , Hunnicutt JL , Xerogeanes JW , Woolf SK , Slone HS . Systematic review of publications regarding quadriceps tendon autograft use in anterior cruciate ligament reconstruction. Arthrosc Sports Med Rehabil. 2019;1:e93–e99. 10.1016/j.asmr.2019.09.001 PMC7120865 32266345

[13] Horstmann H , Petri M , Tegtbur U , Felmet G , Krettek C , Jagodzinski M . Quadriceps and hamstring tendon autografts in ACL reconstruction yield comparably good results in a prospective, randomized controlled trial. Arch Orthop Trauma Surg. 2022;142:281–289. 10.1007/s00402-021-03862-8 PMC8783919 33742222

[14] Howell SM , Clark JA , Blasier RD . Serial magnetic resonance imaging of hamstring anterior cruciate ligament autografts during the first year of implantation. A preliminary study. Am J Sports Med. 1991;19:42–47. 10.1177/036354659101900107 2008929

[15] Kanda Y . Investigation of the freely available easy‐to‐use software “EZR” for medical statistics. Bone Marrow Transpl. 2013;48:452–458. 10.1038/bmt.2012.244 PMC3590441 23208313

[16] Kinoshita T , Hashimoto Y , Iida K , Nakamura H . ACL graft matching: cadaveric comparison of microscopic anatomy of quadriceps and patellar tendon grafts and the femoral ACL insertion site. Am J Sports Med. 2022;50:2953–2960. 10.1177/03635465221110895 35914183

[17] Komzák M , Hart R , Náhlík D , Vysoký R . In vivo knee rotational stability 2 years after the ACL reconstruction using a quadriceps tendon graft with bone block and bone‐patellar tendon‐bone graft. Arch Orthop Trauma Surg. 2022;142:1995–1999. 10.1007/s00402-021-04195-2 34601649

[18] Korcari A , Przybelski SJ , Gingery A , Loiselle AE . Impact of aging on tendon homeostasis, tendinopathy development, and impaired healing. Connect Tissue Res. 2023;64:1–13. 10.1080/03008207.2022.2102004 PMC9851966 35903886

[19] Krutsch W , Szymski D , Rüther J , Musahl V , Grassi A , Tischer T , et al. Sport‐specific differences in ACL injury, treatment and return to sports: Football. Knee Surg Sports Traumatol Arthrosc. 2025;33:4050–4058. 10.1002/ksa.12803 PMC12582233 40814850

[20] Kunze KN , Moran J , Polce EM , Pareek A , Strickland SM , Williams RJ . Lower donor site morbidity with hamstring and quadriceps tendon autograft compared with bone‐patellar tendon‐bone autograft after anterior cruciate ligament reconstruction: a systematic review and network meta‐analysis of randomized controlled trials. Knee Surg Sports Traumatol Arthrosc. 2023;31:3339–3352. 10.1007/s00167-023-07402-2 37000243

[21] Kwan KYC , Ng KWK , Rao Y , Zhu C , Qi S , Tuan RS , et al. Effect of aging on tendon biology, biomechanics and implications for treatment approaches. Int J Mol Sci. 2023;24:15183. 10.3390/ijms242015183 PMC10607611 37894875

[22] Lansdown DA , Riff AJ , Meadows M , Yanke AB , Bach BR . What factors influence the biomechanical properties of allograft tissue for ACL reconstruction? A systematic review. Clin Orthop Relat Res. 2017;475:2412–2426. 10.1007/s11999-017-5330-9 PMC5599386 28353048

[23] Lazarides AL , Alentorn‐Geli E , Vinson EN , Hash TW , Samuelsson K , Toth AP , et al. Advanced patellar tendinopathy is associated with increased rates of bone‐patellar tendon‐bone autograft failure at early follow‐up after anterior cruciate ligament reconstruction. Orthop J Sports Med. 2018;6:2325967118807710. 10.1177/2325967118807710 PMC6243419 30480020

[24] Matsuo T , Kusano M , Uchida R , Tsuda T , Toritsuka Y . Anatomical rectangular tunnel anterior cruciate ligament reconstruction provides excellent clinical outcomes. Knee Surg Sports Traumatol Arthrosc. 2022;30:1396–1403. 10.1007/s00167-021-06609-5 34014338

[25] Meena A , Farinelli L , D'Ambrosi R , Runer A , Attri M , Rudraraju RT , et al. Both hamstring and quadriceps tendon autografts offer similar functional outcomes after arthroscopic anterior cruciate ligament reconstruction in patients aged 50 years or older. Arthroscopy. 2025;41:1512–1520. 10.1016/j.arthro.2024.06.044 38992514

[26] Nuelle CW , Shubert D , Leary E , Pringle LC . Two‐dimensional magnetic resonance imaging in preparation for autograft anterior cruciate ligament reconstruction demonstrates quadriceps tendon is thicker than patellar tendon. Arthrosc Sports Med Rehabil. 2023;5:e783–e791. 10.1016/j.asmr.2023.04.005 PMC10300585 37388871

[27] Nyland J , Collis P , Huffstutler A , Sachdeva S , Spears JR , Greene J , et al. Quadriceps tendon autograft ACL reconstruction has less pivot shift laxity and lower failure rates than hamstring tendon autografts. Knee Surg Sports Traumatol Arthrosc. 2020;28:509–518. 10.1007/s00167-019-05720-y 31538227

[28] Offerhaus C , Albers M , Nagai K , Arner JW , Höher J , Musahl V , et al. Individualized anterior cruciate ligament graft matching: in vivo comparison of cross‐sectional areas of hamstring, patellar, and quadriceps tendon grafts and ACL insertion area. Am J Sports Med. 2018;46:2646–2652. 10.1177/0363546518786032 30059247

[29] Raj S , Ridha A , Searle HKC , Khatri C , Ahmed I , Metcalfe A , et al. Quadriceps tendon versus hamstring tendon graft for primary anterior cruciate ligament reconstruction: a systematic review and meta‐analysis of randomised trials. Knee. 2024;49:226–240. 10.1016/j.knee.2024.07.002 39043018

[30] Runer A , Csapo R , Hepperger C , Herbort M , Hoser C , Fink C . Anterior cruciate ligament reconstructions with quadriceps tendon autograft result in lower graft rupture rates but similar patient‐reported outcomes as compared with hamstring tendon autograft: a comparison of 875 patients. Am J Sports Med. 2020;48:2195–2204. 10.1177/0363546520931829 32667271

[31] Schweitzer ME , Mitchell DG , Ehrlich SM . The patellar tendon: thickening, internal signal buckling, and other MR variants. Skeletal Radiol. 1993;22:411–416. 10.1007/BF00538442 8248814

[32] Shelton TJ , Delman C , McNary S , Taylor JR , Marder RA . Aging decreases the ultimate tensile strength of bone‐patellar tendon‐bone allografts. Arthroscopy. 2021;37:2173–2180. 10.1016/j.arthro.2021.02.042 33713755

[33] Shumborski S , Salmon LJ , Monk C , Heath E , Roe JP , Pinczewski LA . Allograft donor characteristics significantly influence graft rupture after anterior cruciate ligament reconstruction in a young active population. Am J Sports Med. 2020;48:2401–2407. 10.1177/0363546520938777 32736505

[34] Takeuchi S , Rothrauff BB , Taguchi M , Kanto R , Onishi K , Fu FH . In situ cross‐sectional area of the quadriceps tendon using preoperative magnetic resonance imaging significantly correlates with the intraoperative diameter of the quadriceps tendon autograft. Knee Surg Sports Traumatol Arthrosc. 2021;29:742–749. 10.1007/s00167-020-05993-8 32333056

[35] Yamasaki S , Hashimoto Y , Iida K , Han C , Kinoshita T , Nishino K , et al. Quadriceps tendon with bone autograft has better stability and magnetic resonance imaging maturation than hamstring tendon autograft after anterior cruciate ligament reconstruction in patients with knee hyperextension. Arthroscopy. 2024;40:1234–1244. 10.1016/j.arthro.2023.08.013 37597704

[36] Yu JS , Popp JE , Kaeding CC , Lucas J . Correlation of MR imaging and pathologic findings in athletes undergoing surgery for chronic patellar tendinitis. Am J Roentgenol. 1995;165:115–118. 10.2214/ajr.165.1.7785569 7785569

[37] Zuskov A , Freedman BR , Gordon JA , Sarver JJ , Buckley MR , Soslowsky LJ . Tendon biomechanics and crimp properties following fatigue loading are influenced by tendon type and age in mice. J Orthop Res. 2020;38:36–42. 10.1002/jor.24407 PMC6917867 31286548

